# Top-Down and Bottom-Up Approaches to Environmental Governance in China: Evidence from the River Chief System (RCS)

**DOI:** 10.3390/ijerph17197058

**Published:** 2020-09-27

**Authors:** Jie Ouyang, Kezhong Zhang, Bo Wen, Yuanping Lu

**Affiliations:** 1 School of Management, Huazhong University of Science and Technology, Wuhan 430074, China; jie_ouyang@hust.edu.cn (J.O.); zkzdr@mail.hust.edu.cn (K.Z.); 2 Department of Public Policy, City University of Hong Kong, Hong Kong, China; 3 School of Public Finance and Taxation, Zhongnan University of Economics and Law, Wuhan 430070, China; yuanpinglu@zuel.edu.cn; 4 Institute of Income and Public Finance, Zhongnan University of Economics and Law, Wuhan 430070, China

**Keywords:** river chief system (RCS), top-down bureaucratic structure, bottom-up citizen participation, China’s environmental governance regime, policy implementation and effectiveness, water environment and pollution control

## Abstract

A common argument is that the comprehensive implementation of the river chief system (RCS) is a clear indication of the Chinese government’s strong commitment to overcoming the problem of water pollution. Scant attention, nonetheless, has been afforded to systematically examining the economic and social effects of this pioneering policy. Based on news reports and data from regions in which the RCS was piloted, this paper fills in a critical literature gap by unpacking the environmental, economic, and societal benefits accrued from this river-based management approach. Specifically, by employing a difference-in-differences (DID) method, this study shows that (1) overall, the adoption of the RCS has significantly reduced the discharge of sewage per unit of GDP and improved water quality to a considerable extent; (2) the RCS, functioning under China’s top-down bureaucratic structure, coupled with increasing encouragement of bottom-up oversight and citizen participation, has provided local governments with strong incentives to improve water quality in a timely manner in their respective jurisdictions through the introduction of a plethora of measures, ranging from increased investment in wastewater treatment to faithful enforcement of environmental regulations; (3) the positive changes anticipated as a result of the RCS cannot be materialized in regions that have difficulties sustaining economic growth or facilitating cross-boundary policy coordination; and (4) the long-term effectiveness of the RCS is based on its ability to compel local enterprises to innovate their modes of operation, ultimately leading to regional industrial upgrading. The paper concludes by discussing how these empirical findings can help policymakers devise feasible tactics for confronting the causes of China’s current environmental predicament in the context of improving the alignment of individual officials’ political aspirations with targeted environmental outcomes.

## 1. Introduction

Forging solutions to effectively curtail the pollution of rivers and lakes has become an essential governance task that must be accomplished by the majority of developing countries. China is a prominent case in point. Over the past four decades, China has experienced miraculous economic growth by virtue of its opening-up reform, which has concomitantly been accompanied by problems such as severe environmental degradation and water pollution [1,2]. Although the central government has made concerted efforts to redress environmental harm by enacting several environmental laws and regulations, water pollution control has remained subpar and, hence, widely regarded as a major roadblock to the nation’s sustainable development [3]. According to China’s Environmental Conditions Report released by the then Ministry of Environmental Protection in 2016, out of seven main waster systems, five were considered polluted to various degrees. In addition, among samples from 6124 sites from which the respective underground water was sampled for examination, over 60% failed to fulfill the minimum threshold for quality. These statistics bear real-world consequences and social repercussions [4]. As asserted by the Chinese Academy of Engineering in 2011, the wellness of an estimated 300 million individuals was being threatened by diseases originating from polluted water; specifically, one-third of the water being consumed barely fulfilled the national drinking-water standard, and one-fifth of arable lands was contaminated by heavy metal pollutants [5]. Several newsworthy incidents on a societal scale triggered by water pollution, ranging from an outbreak of the blue-green algae on Lake Tai in 2007, dead pigs floating on the Huangpu River in 2013, to highly carcinogenic sewage draining directly into the Yangtze River in 2015, have undoubtedly added fuel to the fire and subsequently strengthened the central government’s resolve to address water contamination at its root.

In December 2016, the Central Committee of the Chinese Communist Party and the State Council co-published an official document, “Opinions on Promoting the River Chief System,” which explicitly required a river chief system (RCS) at all provincial, municipal, county, and township levels to be established by the end of 2018. Under the RCS scheme, Party secretaries and government leaders at various administrative levels jointly serve as river chiefs in their respective jurisdictions and share the responsibilities of water environmental management, the fulfillment of which counts as a key performance indicator for promotion-hopeful officials. For instance, river chiefs face dismissal from their leadership positions if major water pollution-related incidents occur in their governing jurisdictions. A new governance model for water pollution has therefore been launched.

In retrospect, the reasons underlying the central authorities’ predilection toward and subsequent mandatory diffusion of the RCS can in large part be ascribed to the satisfactory results achieved in Wuxi. Initially promoted and implemented by the Wuxi municipal government, the RCS was considered no more than a local trial of an innovative environmental governance strategy. Nonetheless, it rose to fame by immensely helping the Wuxi Municipal Government, which faced long-standing difficulties in the local implementation of environmental regulations, strengthened the performance of water pollution control, clarified the environmental responsibilities among various integral departments, and established a coordination mechanism based on future water-related challenges [6,7]. More notably, the RCS attracted public participation, which is increasingly considered an indispensable ingredient of effective local environmental governance. Under the RCS scheme, for example, the Wuxi government not only hired local residents to routinely monitor the amount of sewage discharge but also set up information boards on which the contact details of river chiefs and general complaint hotlines were publicized in various conspicuous places along the riverbank [8]. Owing to the fact that the RCS as a policy instrument is now being steadfastly drawn on by other municipalities in China and has recently been institutionalized by the central government as a must-have component of local environmental regulatory regimes, this paper investigates the generalizability of the Wuxi experience, assessing whether the RCS setup, which simultaneously reflects the interplay processes of China’s bottom-up and top-down environmental management, is effective in curbing the problems of local water pollution.

Early scholars with limited understanding of the complexity of pollution problems generally believed that the resolution of environmental problems only required top-down executive orders and controls [9,10]. In the context of China, this idea has proven partially effective at best [11]. Confined in a highly centralized personnel system, local cadres (i.e., political elites staffing China’s huge party–state apparatus at the local level, see: [12]) are motivated to abide by the environmental orders issued by their superior governments, leading to immediate improvement in water pollution management [13]. However, “this top-down mindset alone neither works well with complex problems nor in the long run” [10] (p. 573). Due to inevitable information asymmetry, national decision-makers are often unable to fully consider regional heterogeneity [14], resulting in the formulation of one-size-fits-all policies and regulations. Moreover, scholars have contended that arranging top-down monitoring and supervision in a gigantic, hierarchical pyramid is prohibitively costly and inefficient, causing the proliferation of implementation gaps and fraudulent environmental data reporting from below [15,16]. To further complicate the situation, a top-down approach precludes the possibility of engaging local stakeholders and may even be subject to objections and resentment on the part of local residents if certain dimensions of state-mandated measures are unfavorably interpreted [17,18].

Admittedly, another stream of research has increasingly advocated for a decentralized and participatory governance mode to achieve environmental policy objectives in a more sustainable and effective manner [19,20]. In essence, approaches that reflect this ideology have often been characterized as “bottom-up” because they attend heavily to local interests and search for context-adaptive solutions to local environmental problems. Unfortunately, although bottom-up regimes have been widely employed by major developed countries to address environmental problems, a particular challenge in practice arises as local activities and wisdom are difficult to coordinate; thus, problems with negative externalities and/or regional spillover are unlikely to be collectively solved with ease [8,21]. Moreover, local environmental administrations and officials are more susceptible to lobbying by strong interest groups [22]. The resulting regulatory capture is bound to put the public welfare in jeopardy.

If neither a top-down nor bottom-up approach alone is capable of resolving China’s current environmental predicaments, the possibility of combining the two modes must be envisaged such that their respective strengths and weaknesses can complement each other. According to Riker [23], the actual effects of a decentralized governance structure hinge on the country’s degree of political centralization [24]. This belief indicates that the integration of top-down and bottom-up policymaking and implementation approaches could be of immense utility [10,25]. Specifically, a bottom-up approach makes fuller use of local information and encourages policy innovation. Nonetheless, in authoritarian states such as China, the incentives of local players are largely shaped by higher-level governments. In this regard, some elements of top-down action are also necessary [26] in “offering a sanctioning and coordinating power to frame organizational structures, and in enforcing solutions on decentralized actors to pay heed to spillover pollution” [10] (p. 574).

To our knowledge, other than a handful of single case studies, researchers have not provided empirical evidence of the superiority of governance models integrating both top-down and bottom-up forces in China’s nomenklatura system through which the ruling Communist Party “manages and essentially controls the appointment, promotion, transfer and removal of practically all but lowest ranking officials” [27] (p. 703). This paper, which comprehensively assesses the effects of the RCS on water pollution, serves as an ideal point of departure to fill this research gap. By evaluating water pollution statistics from 113 Chinese cities between 2004 and 2014, the difference-in-differences (DID) method is used as the main identification strategy to gauge RCS effectiveness. It turns out that (1) the inception and implementation of the RCS motivated local governments to increase both their investments in water pollution control and stringency in enforcing environmental regulations, leading to improved water quality; (2) the success of the RCS has depended to a large extent on whether the cities adopting this system could maintain a stable rate of economic growth and facilitate smooth coordination with neighboring jurisdictions; and (3) from a long-term perspective, the impact of the RCS lies in its ability to accelerate the pace of firms’ innovation, which is a requisite condition for industrial upgrading and economic restructuring that are key to China’s future competitiveness and economic viability.

The remainder of this paper is organized as follows: The institutional background and theoretical framework are detailed in Section 2. Section 3 presents the development of the research hypotheses, which are investigated in line with the narrative description of data sources and model specification in Section 4. Empirical findings are presented in Section 5, followed by robustness checks demonstrating the validity of the main findings. The paper concludes by summarizing the structural and institutional factors contributing to the current success of RCS adoption, acknowledging major limitations of the paper, and offering policy implications that enable public leaders to make the most of the RCS in their preparation for future hurdles brought about by local water governance.

## 2. Institutional Background and Theoretical Framework

The introduction of the RCS is a manifestation of institutional exploration and innovation on the part of local Chinese governments. In August 2007, in response to the urban water supply crisis caused by large-scale cyanobacterial blooms in Lake Tai, the Wuxi municipal government issued an official document, “Decisions on Establishing the River (Lake, Reservoir, Creek, and Spring) Chief System and Forging a Comprehensive Governance System Managing Lakes, Reservoirs, Creeks, and Springs.” In addition to detailing phase-based objectives, supporting institutions, the chain of accountability, and performance assessment rubrics and methods, this document explicitly required the government and party leaders (heads or acting heads) of county-level cities subordinate to the Wuxi municipality to become “river chiefs” and shoulder water governance and pollution control responsibilities in their respective jurisdictions. Inspired by the Wuxi approach, the Jiangsu provincial government promoted the RCS in the governance of the entire Lake Tai basin in the following year. Specifically, all 15 rivers flowing into Lake Tai were subject to the “dual-RCS,” in which government leaders at both the municipal and provincial levels were appointed as co-stewards for each waterway. In less than 4 years, the RCS was implemented in all 13 municipal-level cities in Jiangsu Province, elevating the significance of RCS adoption from the achievement of international standards for water quality to attainment of a river-oriented integrative governance structure that guarantees ecological, water supply, and flood control safety.

In its quest for a more effective model of water environmental governance, Zhejiang began to pilot the RCS in 2008 in several of its subordinate municipalities, including (in chronological order) Huzhou (Changxing County), Jiaxing, Wenzhou, and Jinhua. In 2013, an official announcement of full territorial coverage of the RCS was made by the Zhejiang provincial government, whose deep belief in the effectiveness of the RCS became palpable. Subsequently incorporated into a governance framework of “simultaneously managing five waters”—regulating water pollution, preventing floodwater damage, discharging drainage water, stabilizing the clean water supply, and promoting water conservation—the RCS experienced smooth and wide diffusion in the region. More notably, Jiangsu and Zhejiang were only two cases in point regarding RCS adoption. Kunming’s renewed management of Dian Lake, Hebei’s comprehensive governance plan for Ziya River, Liaoning’s all-basin governance initiative, Guangdong’s incremental river-governance strategy, and many other examples have corroborated the growing pervasiveness of the RCS. According to the Opinions on a Full Implementation of the River Chief System, jointly issued by the General Offices of the Communist Party and the State Council, as of the end of 2016, the RCS had been holistically implemented and partially piloted in 8 and 16 Chinese provinces, respectively.

### 2.1. Institutional Background of China’s Environmental Governance

As documented in the voluminous literature, China’s environmental governance has been fraught with local noncompliance and malfeasance in the wake of highly salient and omnipresent administrative reforms implemented under a decentralized authoritarian regime over the past decades [28,29]. Despite the differences in their intended objectives, those reforms were unvaryingly obsessed with detailing economic development targets, oftentimes in the form of GDP figures, that local governments were expected to fulfill by using all their discretionary power and resources [30,31,32]. Although undeniably contributing to local economic prosperity, this one-dimensional GDP growth-oriented mindset on the part of the top leadership had unintendedly undermined local officials’ commitment to environmental protection and subsequently led to the emergence of a plethora of environmental problems [14,33,34,35].

Admittedly, China’s central authorities have begun to implement a series of measures to “harmonize economic and social development with environmental protection” [36] (p. 434). The actual effects of these environmentally-conscious approaches, however, have been unsatisfactory [37,38]. Many academics have attributed such a frustrating disconnect to China’s Tiao-Kuai administrative structure [39], which epitomizes “the internal division of power within the Chinese Party-state” [40] (p. 3). Specifically, “vertical bureaucratic relationships linking central to local organizations are commonly referred to as Tiao, while horizontal bodies coordinating actions within given geographic areas are known as Kuai” [40] (pp. 3–4). For example, a municipal-level water affairs bureau in China is subject to the dual leadership of both functional and territorial superiors; namely, its provincial counterpart and the municipal government of the jurisdiction in which the bureau is geographically located, respectively. The resulting cross-hatch of authorities, unfortunately, put this Water Affairs Bureau in the difficult position of having to cater to the heterogeneous, oftentimes conflicting, preferences of its “vertical” and “horizontal” masters. That is, while taking environmental-related orders from above, the bureau had to simultaneously maintain vigilance to the “GDP-centered zealousness” of local government that holds strong control over budgetary allocations and personnel assignments of all public bureaucracies in the region [41] (p. 347). Succumbing to strong parochial interests that prioritize economic goals over other governance tasks, local environmental entities have thus been relaxing their enforcement of environmental laws and regulations, leading to an implementation failure widely known to be the root cause of China’s continuing environmental deterioration [5,42,43].

In addition, the notoriously fragmented and overlapping nature of China’s environmental governance structure is another significant factor hampering the effective local implementation of environmental policies in China [36,44]. In the realm of environmental protection, fragmented environmental bureaucracies have mainly resulted in apparent difficulties in interdepartmental coordination. As vividly put by Kostka [45] (p. 22):

“The implementation and enforcement of environmental mandates at the local level is partly hindered by the fragmented and ambiguous allocation of environmental responsibilities. Usually, numerous government agencies are responsible for the implementation of a single environmental issue but sometimes without a clear division of labor, which in practice ultimately leads to a lack of accountability (Ran, 2013). For example, more than five departments have a role to play in energy efficiency implementation at subnational levels: the local Development and Reform Commission (DRC), the Economic Commission, the Construction Department, the Transportation Department, and the Environmental Protection Bureau (EPB).”

### 2.2. A Theoretical Account of the Influence of the RCS on the Abatement of Water Pollution

**Figure 1:**
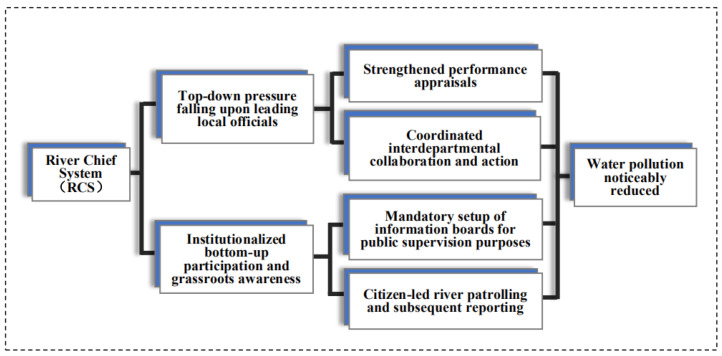
Theoretical Framework.

Taking into account the aforementioned complex political and bureaucratic landscapes that define the de-facto purview and shape the behavioral incentives of China’s environmental administrations and officials at the local level, it has become apparent that a sheer top-down or bottom-up governance paradigm is unlikely to be an able device to address environmental degradation problems in a steady fashion [10,26]. Reasoning along this line indicates that a blend of top-down and bottom-up governance efforts, exemplified by the design of the RCS, is probably a more effective method to gradually and sustainably improve China’s environmental conditions, particularly in water environments [46,47]. The overarching theoretical framework regarding the distinctiveness and advantageousness of the RCS is depicted in Figure 1, followed by brief illustrations of its key elements.

First, from a top-down management perspective, compared with the conventional approach in which binding environmental targets are factored into local officials’ annual target-based performance measurement [48], the RCS is better able to forge a holistic and coordinated governance paradigm. To begin with, the RCS lays bare that the government and party leaders are held directly responsible for any environmental negligence or mishaps transpiring in their designated rivers. Given China’s party–state polity, the involvement of commanders-in-chief from both the party and government systems guarantees the legitimacy of and administrative resources necessary for RCS adoption. In addition, the implementation of the RCS has been aided by a strengthened performance evaluation system, which not only has helped align the incentives between central and local administrations, but has also held local cadres unequivocally accountable for their underperformance. More importantly, in China’s “pressurized” political atmosphere, this institutional setup clearly conveys the central intent that the furtherance of the RCS is a priority and its faithful local implementation is expected [34].

Moreover, the RCS adoption successfully circumvents the coordination predicament that has bedeviled China’s environmental systems for years. Primarily due to ambiguous allocation of environmental responsibilities, the Chinese environmental governance regime is commonly characterized as a fragmented colossus; metaphorically speaking, one issue falls under the purview of at least nine departments (*Jiu Long Zhi Shui*), and the communication and coordination mechanisms among these are not formalized and may even be absent. In the scenario of RCS adoption, nonetheless, coordination efforts are minimized because the river chiefs’ role is taken on by party and government heads whose ability to mobilize interdepartmental collaboration is locally unmatched.

**Figure 2:**
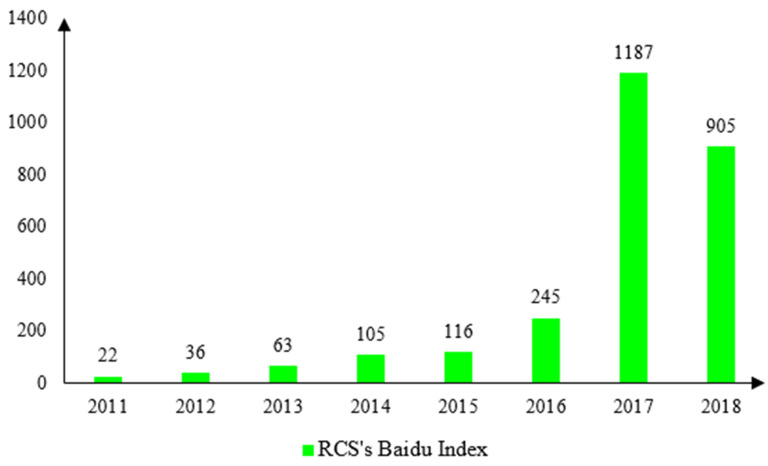
RCS’s Internet Search Index (Baidu Index).

Last but not least, civic engagement and bottom-up activities, judiciously scrutinized and rarely encouraged by governments in their running of local affairs in the not-so-distant past [49], are channeled into the design and furtherance of the RCS. The noticeboards, on which contact details of corresponding river chiefs are publicized, are mandated to be placed at key locations along the river pathways. In some jurisdictions where the RCS was piloted, local residents were hired to “patrol” the rivers so that more precise information could be gleaned to help governments identify pollution sources. More notably, given the novelty of the design of the RCS, it has garnered high-profile media exposure since its inception and subsequently attracted growing citizen participation, enhancing the transparency of the RCS’s work-in-progress implementation. Figure 2 presents the Internet search volume for the RCS in recent years. Based on the Baidu search index, a weighted index that measures China’s netizens’ level of attention paid to keywords of interest, public attention and interest are increasingly afforded to the RCS, particularly when the RCS has begun to roll out across the country since 2016. Admittedly, owing to the paucity of pertinent data, it is difficult for us to provide direct empirical evidence for the bottom-up mechanism. Having said that, we are able to find that there is a significant negative relationship between the Baidu search index concerning the RCS and the amount of wastewater discharge (see the regression Table A1 in the Appendix A). These results combine to show that the citizen-led, bottom-up pressure is very likely to have played a pivotal role in enhancing the effectiveness of the RCS.

## 3. Research Hypotheses

As mentioned, due to the long-standing emphasis on economic growth and neglect of environmental protection [50], the investments made by local governments in pollution control have largely been insufficient. In addition, the ambiguous allocation of environmental responsibilities among functional departments has resulted in the phenomenon of “buck passing” [39], which is a direct cause of the loosely enforced environmental regulations at the local level. In response to these deficiencies, the RCS has made aquatic environmental targets binding in the performance evaluation rubric of local cadres. Local governments and officials, hence, no longer dare to treat environmental issues as “soft targets” that can be ignored in favor of economic growth or be selectively implemented. More importantly, the adoption of the RCS, because of its practical emphasis on “interdepartmental communication and problem-solving,” entails the promise of uprooting the long-standing coordination problems from the ambiguous allocation of water environmental responsibilities [51]. Citizen-led oversight, another powerful supervisory force of local governments, is simultaneously integrated into the design and implementation of the RCS. All these attributes are believed to have collectively brought about improved water conditions. Thus, hypothesis 1 is proposed:

**Hypothesis** **1:** *The RCS has driven local governments to increase investment in water pollution governance and more strictly enforce environmental regulations, leading to improved water quality*.

Striking the delicate balance between economic growth and environmental protection remains a worldwide problem [52,53]. Serving the world’s most populated country, the Chinese government is expected to demonstrate its governance capacity in a multitude of areas, including healthcare, education, employment, and social welfare. The completion of these governance tasks, however, is difficult to attain in the absence of a solid economic basis. This explains the fundamental reason that a cadre evaluation system focusing on GDP growth has been used as the primary incentive tool for local officials over the past four decades in China [31]. Promotion-seeking officials, in this regard, have developed on-the-job principles, which can be characterized by the often-expressed adage: economic growth comes first and environmental matters take a low priority. Today, although the central government has begun to incorporate environmental governance outcomes into the assessment rubric of local officials’ performance, economic development continues to play an insurmountably critical role in the evaluation of overall political achievements delivered by local administrations and officials.

Moreover, severe mismatches between fiscal capacity and expenditure responsibilities on the part of local governments have been widely observed in China [54,55]. Because of the limited financial resources allocated from above, local governments must resort to generating additional revenues from their local economies to maintain daily operations. Consequently, in economically disadvantaged areas where local governments have fewer “opportunities to diversify their economies away from polluting industry” [38], environmental laws and regulations are prone to confront greater challenges and uncertainties in their local implementation practices and outcomes. We therefore postulate that:

**Hypothesis** **2:** *Benefits from the RCS are less likely to be reaped by areas with greater pressure on economic growth*.

Water pollution is notorious for its negative spillover effects, the solution of which requires coordination efforts by all jurisdictions along the route of the waterways of concern [56]. Moreover, environmental regulations can trigger yardstick competition between subnational governments [57,58]. In the absence of a universal implementation plan, cities adopting more stringent environmental laws and regulations are likely to put themselves in a disadvantageous position with respect to attracting investments and liquid capital. Because of their weakened competitiveness in the economic market, these local governments must relax their stringent environmental enforcement, triggering regulatory fiascos across environmental-related matters. Thus:

**Hypothesis** **3:** *The RCS is unlikely to thrive in areas with limited coordination capability. As a corollary, water conditions in those areas are unlikely to improve in the post-RCS era*.

As detailed by Porter and Linde [59], environmental regulations spur innovation because profit-maximizing firms always attempt to offset the cost of compliance by accruing benefits from innovative practices. Properly crafted environmental regulations can thus motivate firms to develop “innovation offsets,” which include finding “less costly materials or better utilization of materials in the process” [59] (p. 103). Following this line of thought, the utility of the RCS adoption is also expected to go beyond the immediate environmental benefits. Theoretically, in order to upgrade and optimize a region’s industrial structure to balance sustainable economic growth and environmental protection, innovation becomes a must. On this note, the long-term impact of the RCS lies in its potential to offer a prime window of opportunity for local governments to encourage technological innovation and adaptation on the part of polluting firms and ultimately promote the upgrading and optimization of the regions’ industrial structure as a whole. Thus, hypothesis 4 is posited as:

**Hypothesis** **4:** *Implementation of the RCS motivates local polluting firms to develop operational innovations*.

## 4. Model Settings, Data Sources, and Variable Descriptions

### 4.1. Model Settings and Variable Descriptions

Because the RCS falls back on an incremental implementation approach through active use of pilot projects, a quasi-experiment can be used to verify its effectiveness in achieving policy objectives. Specifically, the experimental group comprises areas that have adopted the RCS, whereas the control group is made of jurisdictions where the RCS has not yet been employed. Considering that the initial adoption year of the RCS varies across local entities in the experimental group, a dummy variable serving as the DID estimator, *river,* is introduced in which 0 and 1, respectively, denote the pre- and post-RCS stages. The fully specified model in Equation (1) below captures the net impact of the RCS on the improvement in water quality.

(1) $$Y_{\mathrm{it}}\;=\;\beta_{0}\;+\;\beta_{1}{\mathrm{River}}_{\mathrm{it}}{+\alpha \mathrm{Control}}_{\mathrm{it}}+{\;\mu }_{i}+{\;\gamma }_{t}+{\;\varepsilon }_{\mathrm{it}}$$

In Equation (1), $Y_{\mathrm{it}}$ is the dependent variable, representing the extent to which the water environment is polluted. To quantify${\;Y}_{\mathrm{it}}$, we follow the existing literature [60], which measures the severity of water pollution using the natural logarithm of wastewater discharge volume per unit of GDP. Admittedly, water environment pollution can be idiosyncratically measured. To rule out the possibility that the obtained findings are confounded by measurement differences (or errors), alternative indicators are also introduced to measure this dependent variable (Table 1). Results are detailed in the section of robustness checks.

To explain further, ${\mathrm{River}}_{\mathrm{it}}$ is a dummy variable, which equals 1 if region *i* implements the RCS in year *t*, and 0 if otherwise. ${\mathrm{Control}}_{\mathrm{it}}$ is a vector of control variables, including the regions’ (1) economic development phases; (2) industrial structures; (3) population sizes; and (4) degrees of industrial agglomeration. Specifically, the regions’ economic development levels and industrial structures are measured, respectively, by the natural log form of local GDP (ln GDP) and proportions of the secondary industry (ln GDP_2). Similarly, the population scale is expressed as the natural log of the region’s total population (ln population). The degrees of regional industrial agglomeration (ln IA) are calculated using the number of “above-scale” firms divided by the geographic size of the administrative areas.

Finally, $\mu_{i}$ denotes the fixed-effect at the municipal level, ${\;\gamma }_{t}$ marks the time fixed-effect, and ${\;\varepsilon }_{\mathrm{it}}$ is the error term in which all unobserved factors influencing the water environment of the discussed areas are contained. This article focuses primarily on the coefficient of${\;\mathrm{River}}_{\mathrm{it}}$, namely $\beta_{1}.\;$Holding all other factors constant, $\beta_{1}$ reflects the net effect of the RCS policy on the abatement of water pollution. A descriptive analysis of all the aforementioned variables is provided in Table 2.

### 4.2. Data Sources

To conduct empirical analysis, this paper relied on a panel data set between 2004 and 2014 for 113 municipalities, officially termed “key environmental protection cities” by the then Ministry of Environmental Protection of China. Our panel data set contains information publicly available in the “China Environmental Yearbook” concerning the levels of each city’s industrial wastewater discharge and investment in water pollution governance. Patent data at the regional level were retrieved from the official website of the Chinese Patent Office. In addition, because the implementation status of the RCS is the variable of utmost importance in this paper, each jurisdiction’s RCS implementation status in each year was manually gleaned from policy documents and news reports on the adoption of the RCS at the provincial, municipal, and autonomous region levels. Other macro-level data, including regions’ GDPs, industrial structures, and population sizes, were obtained directly from the “China City Statistical Yearbook”.

Moreover, to study the micro-level impact of the adoption of the RCS on private enterprises, this paper also uses data from the Chinese Private Enterprise Survey (2010–2012). This survey is jointly conducted by the United Front Work Department of the Chinese Communist Party, the All-China Federation of Industry and Commerce, and the China Institute of Private Economy Research every other year by soliciting opinions from business leaders in different sectors nationwide. Widely used in some highly cited research [61,62], this survey is of a desirable level of sample representativeness and offers an informative glimpse into the environmental-related investments and innovation efforts on the part of private businesses.

## 5. Empirical Findings

### 5.1. Effects of the RCS on Water Pollution Governance

Regression results concerning the first hypothesis are reported in Table 3. In addition to simultaneously controlling for time and city-level fixed effects, the approach in Kahn et al. (2015) is used to cluster the standard errors at the province–year level to mitigate the effects of potential heteroscedasticity and spatial correlation. The first row presents the effect of RCS adoption on local governments’ financial spending on water environmental governance. The slope coefficient of River_it_ has a positive sign, indicating that a greater proportion of government expenditures are indeed allotted to treat wastewater since RCS adoption. Specifically, column (1) indicates that the investment in water pollution control made by governments in the treated group is proportionally 0.3% higher than the mean amount in the control group. Considering that Chinese local governments on average invest 0.7% of their annual revenues in water pollution abatement, a 0.3% increase could also be interpreted as a 43% (0.3%/0.7%) spending surge for water governance alone.

The estimated results listed in column (2) are based on comparisons between microenterprise data and their counterparts extracted from a questionnaire survey of nationwide private enterprises in 2010 and 2012. Notably, the RCS adoption helps strengthen the enforcement stringency of water environmental laws and regulations, because private companies reported paying a significantly higher amount of penalty fees in the post-RCS era. Notably, due to the lack of detailed information on environmental penalty fees generated at the regional level, column (2) displays simple comparative results derived from cross-sectional company data. By putting the slope coefficient of river, 1.629, into context, we are cognizant that the penalty fees paid by enterprises in the experimental group are on average 60% (1.629/2.737, where 2.737 is the aggregated sample mean of penalty charges) higher than that of their control group counterparts.

Column (3) provides further supporting evidence for hypothesis 1. The presence of the RCS is associated with a significant decline in regions’ discharge amounts of industrial wastewater. In other words, with the increasing investments in water pollution governance and increasingly more stringent enforcement of environmental regulations, the overall water environment has improved.

### 5.2. Trade-Off between Economic Growth Maintenance and Environmental Betterment

**Figure 3:**
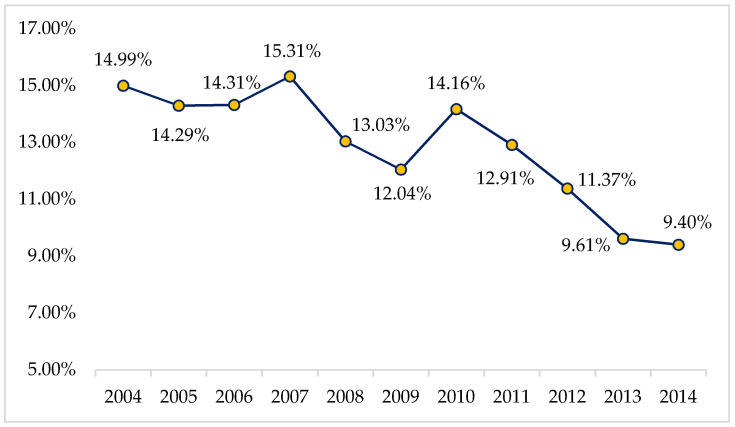
Nominal GDP growth rates from 2004 to 2014 for sample cities (Aggregated). Sample cities refer to the 113 Chinese municipalities, officially termed “key environmental protection cities” by the then Ministry of Environmental Protection of China in 2014.

Depending on location, local governments in China are subject to various levels of pressure resulting from the maintenance of economic growth. In general, local Chinese cities can be classified into two categories: cities with high and low pressure to maintain economic growth. As shown in Figure 3, given that the worldwide financial crisis flared in 2008, China experienced an obvious economic downturn from that point on. By averaging the GDP growth rates of each city in the years of 2008 and 2014, respectively, and subtracting the former from the latter, a base value denoting the average decreasing rate of nationwide GDP growth can be obtained. Therefore, a city is considered a low-pressure city when its declining rate of GDP growth over this time period is less than the national average and a high-pressure city otherwise.

Related regression results are listed in columns (1) and (2) of Table 4. The coefficients of interest indicate that a differential effect exists. For cities with relatively low pressure to maintain economic growth, the adoption of the RCS helps curtail the total discharge amounts of industrial wastewater. However, in cities where the pressure to sustain economic growth is comparatively strong, the significance level of River_it_ declines and the effect size of RCS adoption on the curtailment of wastewater discharge becomes smaller too. More notably, only when data are included for cities ranking in the top quarter of our self-constructed list of economic growth pressure, the slope coefficient of River_it_ becomes statistically insignificant (column 3). In other words, the RCS makes little to no difference to the improvement of water conditions in cities that experienced an above-average decline of economic growth from 2008 to 2014. Hypothesis 2 is thus supported. Apparently, China, in its current stage, continues to manage an inescapable tradeoff between economic growth and environmental protection. Locally sensitive and idiosyncratic governance measures must be devised to avoid placing local administrations in certain localities in the impossible bind of simultaneously maintaining economic growth and faithfully complying with all centrally mandated environmental laws and regulations.

### 5.3. Policy Coordination Across Jurisdictions

To validate the critical role played by the RCS in smoothing interjurisdictional coordination and collaboration, cities in the sample are classified into two categories. Category (a) comprises cities that implemented the RCS in accordance with the arrangement made by their respective provincial governments. More specifically, cities in this category not only adopted the RCS as of the end of 2014 but also served as one of many RCS-adopting cities in their respective provinces (or centrally controlled municipalities). This category includes Zhejiang, Jiangsu, Liaoning, Fujian, and Tianjin. Category (b) comprises cities that implemented the RCS no later than 2014 as a result of their own decision. In other words, these cities were the only RCS-adopting jurisdictions in their respective provinces at the time.

The rationale behind this classification strategy is straightforward: when the decision to adopt the RCS is made at the provincial level, all the cities in that province must comply with this directive. An all-area policy implementation thus precludes the ramifications resulting from interjurisdictional rivalry and/or miscoordination. Local governments do not need to worry excessively about scaring away potential investors or receiving little assistance from neighboring jurisdictions to collectively manage negative externalities generated by water environmental pollution.

The regression results of hypothesis 3 are detailed in columns (4) and (5) of Table 4. As anticipated, the environmental performance of cities in category (a) increases after RCS adoption, evidenced by a significant decline of wastewater discharge per unit of GDP. By contrast, the influence of the RCS on the environmental performance of cities in category (b) is insignificant. These results combine to suggest that the RCS is more likely to be successful in regions where mechanisms coordinating its uniform intraregional implementation are established.

### 5.4. Effect of RCS on Regional Innovation

In column (1) of Table 5 below, the adoption of the RCS has led to greater amounts of research and development (R&D) investments by local firms, whose innovativeness and market values subsequently increase. Additionally, columns (2)–(5) point to the macro-level implications from RCS adoption, affirming a positive relationship between RCS presence and the overall innovation level of the areas concerned (proxied by the number of patents per capita in prefecture-level cities). In summary, consistent with the expectations of hypothesis 4, the adoption of the RCS is empirically proven to have been an impetus for local enterprises to search for innovative means to boost their technical efficiency. If this trend continues, regional industrial upgrading will soon be triggered and the ultimate objective of a long-lasting water environmental governance system can be achieved.

## 6. Robustness Checks

Skeptical readers might voice concerns regarding whether the data used to prove the effectiveness of the RCS in curbing water pollution were “cherry-picked” or if the outcome differences between the experimental and control groups would have existed without the policy intervention. To assuage these concerns and infuse additional confidence in the reliability of the main empirical results, three robustness checks were performed with different time intervals, data sets, and econometric approaches.

### 6.1. Parallel Trend Test: Counterfactual Method

A critical assumption underlying the DID regression is the satisfaction of the parallel trend between the control and experimental groups when the treatment is absent. Following the technique used by Ferrara et al. [63], this paper adopts the counterfactual method to examine whether the parallel trend assumption is fulfilled. Specifically, we start off from an assumption that the adoption of the RCS in each jurisdiction was several years ahead of its actual year of implementation. If the coefficient of *river* under this hypothetical scenario remained negative, the improvement of the water condition at the local level could then be attributed to other unidentified factors in addition to the RCS. Nonetheless, if the coefficient of river became insignificant, this result would indicate no systematic difference in the water condition for both the control and experiment groups in the absence of the RCS.

Table 6 reports the regression results on counterfactual analysis. The core outcome variable of interest, *river,* becomes statistically insignificant when the implementation of the RCS is hypothetically moved forward by 1 to 3 years. The irreplaceable role played by the RCS in curbing wastewater discharge is thus supported.

### 6.2. Reexamination of the Results Using Data from the State-Controlled Monitoring Sites for Water Quality

In China, environmental data publicized by local governments are subject, more or less, to misreporting, embellishment, manipulation, and falsification [15,64]. In the context of RCS implementation, local cadres could be emboldened to change, or at least underreport, the discharge amount of industrial wastewater. This “misconduct” is to prevent themselves from being blamed by the central authorities for their underperformance in water pollution governance and from being subsequently held accountable in the target-based responsibility system. Considering the possibility that the collected data could be imprecise, leading to an overestimation of the impact of the RCS on the curtailment of industrial wastewater discharges, the regression was rerun using the data gathered by the state-controlled monitoring sites for water quality. In this alternative analysis, water pollutant content in rivers and lakes, instead of the amount of sewage discharge, is used as the dependent variable. Data documented by these monitoring sites are credible and useful in their own right primarily because (1) these sites are under the direct command of China National Environmental Monitoring Station and data generated by these sites are thus unlikely to be “distorted” in favor of local governments, and (2) logically, if the RCS helps curtail the discharge of industrial wastewater, the water quality in the concerned areas will improve. The improvement of water quality then further confirms the main empirical findings of the positive utility of the RCS.

Table 7 presents regression results of *river* for corresponding jurisdictions’ water quality assessed by the state-controlled monitoring sites. In columns (1)–(4), RCS adoption noticeably reduces water-pollutant content of various categories in these places. Specifically, rivers and lakes in these locations contain COD, ammonia nitrogen, volatile phenols, and potassium permanganate at significantly lower levels than before adoption, and oxygen is substantially more abundant. Results displayed in column 6 further confirms a non-negligible improvement in water quality in the jurisdictions of interest. Thus, a negative correlation between RCS adoption and the discharge amount of industrial wastewater is observed and consistent among different data source settings.

### 6.3. Reaffirming the Main Results Using the Synthetic Control Method

While the positive influence of the RCS on water pollution governance at the local level has been demonstrated on the basis of the DID and counterfactual approaches elucidated in Section 5.1 and Section 6.1, the causal relationship has not yet been firmly established. In theory, the most ideal design to identify the causal effect of the RCS on water pollution depends on pinpointing an area where the RCS is being implemented. By reasonably guesstimating the current water quality in this area if the RCS was not adopted and subsequently comparing it with the reality in which the RCS has been adopted for years, the impact of the RCS on the improvement of local water quality can be causally captured.

Following this line of thought, the synthetic control method, advocated by scholars such as Abadie and Gardeazabal [65] and Abadie et al. [66], is used to guesstimate the “present” outcome under the assumption that the RCS is absent from the beginning. Specifically, proper weights are assigned to the identified areas where the RCS is not implemented. These weight-laden areas are averaged to form the control group. This constructed control group has two distinct attributes: first, each area is weight adjusted to resemble its counterpart at the pre-RCS stage in the experimental group; second, the post-RCS outcome of each area in the control group exactly mirrors the counterfactual result of its counterpart in the experimental group. In this scenario, the causal influence of the RCS equates to the outcome differences in the post-RCS phase between the synthesized control and experimental groups. Notably, the synthetic control method is based entirely on nonparametric estimation, which extends the scope and therefore verifies the results derived from the DID model. The weights assigned to individual observations in the control group are unvaryingly data driven and auto-generated, alleviating the concerns of endogeneity and selection bias and hence enhancing the credibility of the presented results [67].

**Figure 4:**
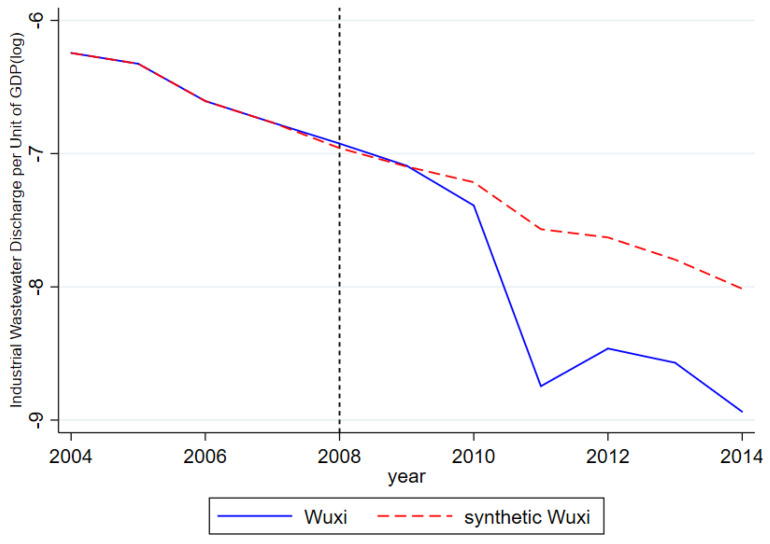
Policy Effects of Wuxi and Synthetic Wuxi.

**Figure 5:**
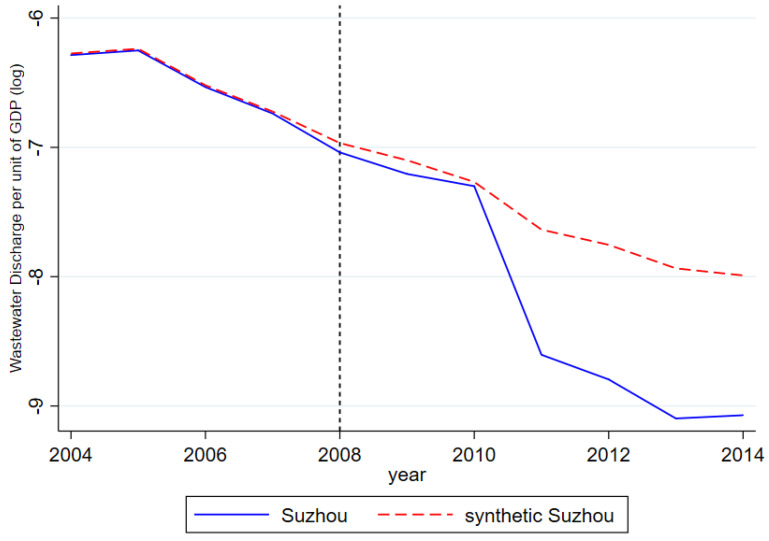
Policy effects of Suzhou and synthetic Suzhou.

Wuxi and Suzhou, two pilot cities of the RCS, are chosen as areas where the synthetic control method is employed to reaffirm the effectiveness of the RCS. As pioneering regions in the adoption of the RCS, Wuxi and Suzhou have accumulated invaluable experiences during its implementation and become the exemplary sites for other cities pursuing similar policies. Table 8 specifies the weights automatically generated for each city when Wuxi and Suzhou are respectively synthetized. The differences in the outcome of interest between the synthetized control and experimental groups are presented in Figure 4 and Figure 5. Based on the result of these simulations, significant declines are observed in the discharged amount of wastewater per unit of GDP in Wuxi and Suzhou after the employment of the RCS. More importantly, noticeable and widening gaps are observed in the wastewater discharge amount between these two cities and their synthesized counterparts. In summary, the adoption of the RCS indeed helps both cities achieve better and more lasting results in water pollution governance.

## 7. Discussion

Navigating effective approaches to remediate and prevent water pollution is a challenging task for developing economies worldwide. By gauging the effectiveness of the RCS, an institutional innovation pioneered by a number of Chinese local governments and subsequently promoted and propagated nationwide, this paper identifies a promising avenue through which water pollution can be more effectively managed in China. From a theoretical standpoint, a dual-track mechanism exemplified by the adoption of the RCS plays a pivotal role in the improvement of the local water environment. Specifically, (1) from a top-down perspective, the authoritarian nature of China’s political system ensures that central directives can be efficiently promoted as mandatory goals that local administrations are expected to fulfill. More importantly, by factoring the curtailment of water pollution into the evaluation of their on-the-job performance, local leading officials become self-motivated to address water-related problems to the best of their abilities for the sake of promotions and other accolades; and (2) from a bottom-up perspective, the emergence of the RCS stems from the exploratory efforts made by local governments, which possess accurate information on their respective localities and are apt to implement tailored measures that remedy local environmental problems. In addition, the noticeboards set up at various spots along riverbanks provide local residents with contact details for the officials in charge of the rivers, inviting citizen participation and oversight. As confirmed by the main regression results of this paper, under this dual-track scheme, the implementation of the RCS indeed induces local government to increase their investments in water pollution control and more resolutely enforce environmental regulations, leading to improved water quality in their respective jurisdictions.

In addition to empirically confirming the positive influence of the RCS on local water environment, this paper contributes to the literature by demonstrating the circumstances under which the utility of the RCS will be greatly compromised, equipping key decision-makers with a more realistic and unvarnished understanding of the long-term prospects of the RCS. The findings indicate that the effects of the RCS in reducing key pollutants of interest are statistically insignificant in jurisdictions with (1) the unremitting pressure of economic growth and (2) difficulties in policy coordination with neighboring jurisdictions. These discoveries are critical in that they have rich implications for the furtherance of the RCS. First, this research demonstrates that the RCS must be strategically promoted in resource-starved jurisdictions in which economic development is naturally prioritized over environmental improvement. For instance, this article determines that RCS adoption spurs local enterprises to innovate their operational practice to be environmental friendly, which leads to increased market competitiveness. Thus, the RCS can be introduced to economically stressed jurisdictions as a stimulus, triggering entrepreneurial innovation and fueling economic growth in the long run. Moreover, supporting institutions that facilitate interjurisdictional collaboration and mediate interregional disputes are needed to materialize (or maximize) the anticipated benefits of the RCS.

This paper has limitations. Theoretically, although this paper showcases that integration of bottom-up and top-down governance approaches are a viable solution to China’s water pollution problems, it fails to empirically validate the effectiveness of the bottom-up processes alone (because of data unavailability) or propose a theoretical framework detailing how this integration can be sustained [68]. Notably, the nationwide implementation of the RCS was proposed against the background of the increasing commitment of the Chinese central government to environmental protection. Whether the RCS will continue to be favorably regarded when the central government in the intermediate future shifts its emphasis from environmental protection back to economic development amid the escalating China—U.S. trade war is unknown. Namely, when environmental performance is deprioritized in year-end evaluations, local cadres are likely to stop focusing on RCS adoption or environmental topics at large. In this scenario, the bottom-up, citizen-led mechanism will be at odds with the motives of promotion-seeking local cadres and subsequently be treated as a nuisance instead of an indispensable supplement of local governance. Empirically, notwithstanding that causal relationships between RCS adoption and the improvement in local water environmental conditions are justified by virtue of a quasi-experimental design, the key data sets used to derive causality abruptly became unavailable in the China Environmental Yearbook from 2014 onward, precluding the possibility of investigating the durability of RCS effectiveness. As the years of the RCS adoption accumulate and more pertinent data become available, the empirical plausibility of our findings can be reexamined. Finally, this paper relies solely on available, secondhand data for analysis, some of which were not collected for academic purposes. As Singleton and Straits [69] (p. 426) contend, “when one uses data collected for another purpose, the search for appropriate data and refinement of the data become extremely important research phases.” Future research can thus incorporate a qualitative component—mainly in the form of semi-structured, in-depth interviews with local officials or enterprise owners—to gather a textured, nuanced, and elaborate understanding of RCS implementation at the local level to capture public awareness and perceptions of the RCS and identify the impetus underlying local citizens’ willingness or unwillingness to participate in the deliberation of environmental topics. Only in this manner can thought-provoking findings be discovered, more well-rounded conclusions drawn, and more actionable policy recommendations offered.

## 8. Conclusions

This paper provides an empirical glimpse into the effectiveness of the RCS in alleviating local water environmental pollution and unpacks the mechanisms whereby the alleged objectives of the RCS are achieved. The analysis suggests that the success of the RCS is attributable to its synthesis of top-down and bottom-up governance approaches—the inherent weaknesses of the former, including prohibitively high information and monitoring costs from the principal–agent relationship, are complemented by the strengths of the latter, whose familiarity with and accessibility to local preferences and information are unmatched. Being cognizant of this fact, decision-makers must more extensively engage social forces, most likely ordinary citizens and nongovernmental entities, to partake in the process of local environmental governance by acting as watchdogs and whistleblowers in their respective jurisdictions, deterring local administrations and officials from paying lip service to environmental matters [70,71]. Additionally, the central government must be well aware that local governments in economically disadvantaged areas are less capable of diversifying their revenue sources and striking a balance between economic growth and environmental protection. That is, RCS adoption cannot proceed in a one-size-fits-all manner and must be strategically customized to accommodate local conditions so that (possible) unintended consequences can be avoided and interregional collaboration forged. Last but not least, to ensure its long-term success, the RCS must be institutionalized and codified. As suggested by Eaton and Kostka [72] (p. 787), although the RCS “appears to be effective in focusing top leaders’ attention on environmental problems, there are also downsides to leaning so heavily on party mechanisms.” The great heed being paid by local government officials to the RCS results from the ruling party’s current emphasis on water environmental governance. If this emphasis wanes or disappears, the fate of the RCS will be in jeopardy. Following this line of thought, advocacy efforts must be launched to institutionalize and legitimize RCS practices to ensure that its local implementation is supported by the rule of law, rather than the whim of top leadership.

## Figures and Tables

**Table 1:** Primary variables and definitions.

Variable Name	Definition
**Panel A: Macrolevel Data Categories—Key Environmental Protection Cities**	
River	River Chief System Implementation Status
Wastewater Discharge	Logarithm of Industrial Wastewater Discharge per Unit of GDP
GDP	Regional Gross Product Logarithm
Population	Total Regional Population Logarithm
GDP_2	Secondary Industry Proportion Logarithm
Industrial Agglomeration (IA)	Degree of Industrial Agglomeration Logarithm
Investment	Proportion of Investment in Sewage Governance to Financial Expenditure
Patent	Total Number of Patents per 10,000 People
Invention Patent	Total Number of Invention Patents per 10,000 People
Utility Model Patent	Total Number of Utility Model Patents per 10,000 People
Industrial Design Patent	Total Number of Industrial Design Patents per 10,000 People
**Panel B: Data Categories Obtained from State-Controlled Monitoring Sites for Water Quality**	
Chemical Oxygen Demand (COD)	COD Content
AD	Ammonia Nitrogen Content
KMno_4_	Potassium Permanganate Content
Volatile Phenol	Volatile Phenol Content
Hg	Mercury Content
DO	Dissolved Oxygen Content
**Panel C: Data Categories Obtained from the Chinese Private Enterprise Survey**	
Penalty	Enterprise Environmental Protection Penalties Logarithm (log (Penalty+1))
Research and Development (R&D)	Enterprise Research and Development Input Logarithm (log (R&D+1))

**Table 2:** Descriptive statistics of major variables.

Variable Name	Observations	Mean	Standard Deviation(s)	Minimum	Maximum
River	1232	0.112	0.316	0	1
Wastewater Discharge (log)	1211	−7.598	0.916	−11.517	−4.637
GDP (log)	1232	16.485	1.027	13.086	19.278
Population (log)	1232	6.015	0.730	3.393	8.124
GDP_2 (log)	1232	3.921	0.224	2.984	4.511
Industrial Agglomeration (log)	1212	−3.359	1.152	−6.501	0.080
Investment	863	0.007	0.010	0	0.099
Patent	763	8.153	19.995	0.091	209.812
Invention Patent	763	2.610	7.956	0.010	92.236
Utility Model Patent	763	2.971	5.796	0.043	66.989
Industrial Design Patent	763	2.572	8.328	0	115.174
COD	1318	4.652	8.961	0.40	177.0
AD	1271	2.124	4.903	0.01	38.70
KMno_4_	1322	5.754	9.913	0.70	195.4
Volatile phenol	1227	0.005	0.014	0	0.203
Hg	1209	0.040	0.142	0	3.080
DO	1341	7.187	1.996	0.50	14.70
Penalty (log (penalty+1))	6220	2.737	4.426	0	19.114
R&D (log (R&D+1))	6132	1.205	2.130	0	10.597

**Table 3:** Effects of the River Chief System (RCS) on water pollution.

	Water Pollution Governance Investment Proportion	Enterprise Payments of Environmental Pollution Fees (Natural logarithm)	Wastewater Discharge per unit of GDP (Natural logarithm)
	(1)	(2)	(3)
River	0.003 *** (0.001)	1.629 *** (0.248)	−0.112 ** (0.0471)
GDP	0.010 ** (0.004)	−0.218 (1.943)	−1.082 *** (0.157)
Population	−0.001 (0.0053)	−0.133 (2.596)	−0.330 (0.198)
GDP_2	−0.004 (0.004)	−8.038 ** (3.459)	−0.263 (0.245)
IA	−0.0004 (0.0014)	−0.149 (0.285)	0.296 *** (0.0438)
Constant	−0.131 ** (0.055)	42.519 (33.430)	14.10 *** (2.167)
Time Effect	YES	YES	YES
City Effect	YES	YES	YES
Sample Size	863	6103	1211
R-Squared	0.496	0.121	0.879

Note: *** and **, indicate the significance levels of 5%, and 10%, respectively; province-year clustered standard errors are in brackets below the coefficients.

**Table 4:** Challenges associated with the implementation of the RCS (partial regression results).

Waste Water Discharge per Unit of GDP (Natural Logarithm)					
	Low Pressure to Maintain Growth	High Pressure to Maintain Growth	Top Quarter Cities with Highest Pressure	Full-Provincial Implementation	Unilateral Implementation
	(1)	(2)	(3)	(4)	(5)
River	−0.130 ** (0.0650)	−0.119 * (0.0608)	−0.047 (0.073)	−0.166 *** (0.0530)	0.0604 (0.0585)
GDP	−1.224 *** (0.190)	−0.859 *** (0.232)	−0.890 *** (0.310)	−1.156 *** (0.289)	−0.985 *** (0.177)
Population	1.292 *** (0.333)	−1.075 *** (0.292)	0.189 (0.568)	9.060 *** (1.292)	−0.6178 ** (0.1896)
GDP_2	−0.333 (0.222)	0.0344 (0.500)	0.682 (0.748)	2.409 *** (0.329)	−0.469 * (0.282)
IA	0.297 *** (0.0444)	0.360 *** (0.0670)	0.439 *** (0.097)	0.384 *** (0.0543)	0.302 *** (0.0485)
Constant	6.936 * (3.598)	13.99 *** (3.127)	4.856 (5.044)	−49.80 *** (7.074)	15.877 *** (2.229)
Time Effect	YES	YES	YES	YES	YES
City Effect	YES	YES	YES	YES	YES
Sample Size	637	574	299	267	944
R-Squared	0.887	0.878	0.856	0.885	0.892

Note: ***, **, and * indicate the significance levels of 1%, 5%, and 10%, respectively; province-year clustered standard errors are in brackets below the coefficients.

**Table 5:** Effects of the RCS on regional innovation.

	Enterprise R&D Inputs (Natural Log)	Total Number of Patents	Total Number of Invention Patents	Total Number of Utility Model Patents	Total Number of Design Patents
	(1)	(2)	(3)	(4)	(5)
River	0.374 *** (0.092)	13.26 ** (5.945)	3.012 ** (1.427)	2.508 * (1.287)	7.741 ** (3.700)
GDP	−0.127 (0.843)	−3.708 (5.210)	−0.479 (2.219)	−3.820 ** (1.641)	0.591 (2.320)
Population	−0.0812 (0.7187)	26.95 *** (8.762)	10.56 ** (4.669)	11.89 *** (3.159)	4.499 ** (1.889)
GDP_2	−2.788 ** (1.038)	−52.83 *** (11.64)	−20.14 *** (4.678)	−14.40 *** (2.461)	−18.29 *** (6.605)
IA	0.142 * (0.073)	−1.975 * (1.097)	−0.508 * (0.303)	−0.649 ** (0.295)	−0.818 (0.625)
Constant	15.770 (12.019)	100.3 (61.12)	23.18 (27.56)	47.05 ** (21.51)	30.02 (25.57)
Time Effect	YES	YES	YES	YES	YES
City Effect	YES	YES	YES	YES	YES
Sample Size	6015	759	759	759	759
R-Square	0.118	0.797	0.856	0.820	0.623

Note: ***, **, and * indicate the significance levels of 1%, 5%, and 10%, respectively; province-year clustered standard errors are in brackets below the coefficients.

**Table 6:** Parallel trend test: counterfactual experiment.

	Waste Water Discharge per Unit of GDP (Natural Log)		
	One Year Prior	Two Year Prior	Three Year Prior
	(1)	(2)	(3)
River	−0.0655 (0.0472)	−0.00678 (0.0472)	0.0722 (0.0484)
GDP	−1.077 *** (0.157)	−1.073 *** (0.158)	−1.068 *** (0.158)
Population	−0.325 (0.197)	−0.324 * (0.196)	−0.333 * (0.195)
GDP_2	−0.262 (0.247)	−0.241 (0.248)	−0.197 (0.251)
IA	0.295 *** (0.0441)	0.292 *** (0.0443)	0.291 *** (0.0443)
Constant	13.96 *** (2.179)	13.76 *** (2.187)	13.50 *** (2.192)
Time Effect	YES	YES	YES
City Effect	YES	YES	YES
Sample Size	1211	1211	1211
R-Squared	0.879	0.879	0.879

Note: *** and * indicate the significance levels of 1% and 10%, respectively; province-year clustered standard errors are in brackets below the coefficients.

**Table 7:** Effects of the RCS on water quality (data gleaned from state-controlled monitoring sites).

	COD Content	Ammonia Nitrogen Content	Potassium Permanganate Content	Volatile Phenol Content	Mercury Content	Dissolved Oxygen Content
	(1)	(2)	(3)	(4)	(5)	(6)
River	−5.326 *** (1.366)	−0.678 ** (0.297)	−7.708 *** (1.358)	−0.009 *** (0.002)	−0.089 (0.056)	1.105 *** (0.207)
GDP	2.822 * (1.646)	−0.830 (1.280)	−0.652 (1.574)	−0.002 (0.004)	0.041 (0.038)	−0.268 (0.334)
Population	3.672 (5.364)	−0.130 (1.554)	5.289 (3.696)	−0.002 (0.011)	−0.003 (0.171)	−0.856 (0.853)
GDP_2	−3.530 (2.865)	0.251 (0.568)	−3.794 (2.537)	0.007** (0.003)	0.002 (0.070)	0.0756 (0.321)
IA	1.071 * (0.603)	0.542 ** (0.244)	1.158 * (0.606)	0.0002 (0.002)	0.014 (0.010)	−0.111 (0.102)
Constant	−5.326 *** (1.366)	−0.678 ** (0.297)	−7.708 *** (1.358)	−0.009 *** (0.002)	−0.089 (0.056)	1.105 *** (0.207)
Time Effect	YES	YES	YES	YES	YES	YES
Monitoring Site Effect	YES	YES	YES	YES	YES	YES
Sample Size	1318	1271	1322	1227	1209	1341
R-Squared	0.694	0.884	0.698	0.645	0.476	0.858

Note: ***, ** and * indicate the significance levels of 1%, 5%, and 10%, respectively; province-year clustered standard errors are in brackets below the coefficients. The explanatory variable in the table is the water pollution content observed at each test site.

**Table 8:** City weights of synthetic Wuxi and Suzhou.

City Weight of Synthetic Wuxi and Suzhou								
City Name	Wuxi	Suzhou	City Name	Wuxi	Suzhou	City Name	Wuxi	Suzhou
Anyang	0	0.008	Lanzhou	0	0.006	Wuhu	0	0.008
Baotou	0	0.004	Linfen	0	0.01	Wuhan	0	0.006
Baoding	0	0.008	Liuzhou	0.054	0.112	Xi’an	0	0.01
Baoji	0	0.008	Luoyang	0	0.006	Xining	0	0.015
Beihai	0	0.012	Luzhou	0.094	0.013	Xianyang	0	0.008
Beijing	0	0.003	Ma’anshan	0	0.007	Xiangtan	0.054	0.01
Changde	0.001	0.011	Mianyang	0	0.009	Yanan	0	0.005
Chongqing	0	0.01	Mudanjiang	0	0.012	Yangquan	0	0.006
Changchun	0	0.007	Nanchang	0	0.008	Yibin	0	0.009
Changsha	0	0.004	Nanning	0	0.008	Yichang	0	0.01
Changzhi	0	0.008	Panzhihua	0	0.005	Yinchuan	0	0.011
Chengdu	0.058	0.008	Pingdingshan	0	0.007	Yueyang	0	0.009
Chifeng	0	0.006	Qinhuangdao	0	0.008	Zaozhuang	0	0.008
Daqing	0	0.006	Qingdao	0	0.005	Zhanjiang	0	0.007
Datong	0	0.007	Rizhao	0	0.01	Zhangjiajie	0	0.007
Guilin	0	0.007	Sanya	0	0.007	Zhengzhou	0	0.007
Guiyang	0	0.006	Shantou	0.482	0.008	Zhongshan	0	0.008
Haikou	0	0.003	Shanghai	0	0.005	Zhuhai	0	0.007
Huhhot	0	0.011	Shaoguan	0.257	0.025	Zhuzhou	0	0.008
Jilin	0	0.327	Shizuishan	0	0.006	Zhunyi	0	0.005
Jining	0	0.006	Tai’an	0	0.005	Urumqi	0	0.007
Jiaozuo	0	0.009	Taiyuan	0	0.005	Karamay	0	0.005
Jinchang	0	0.006	Tangshan	0	0.008			
Jingzhou	0	0.011	Tongchuan	0	0.005			
Jiujiang	0	0.008	Weihai	0	0.004			
Kaifeng	0	0.007	Weifang	0	0.007			

## Appendix A

**Table A1:** The effects of bottom-up pressure on water pollution.

	Wastewater Discharge per Unit of GDP (Natural Log)	
	(1)	(2)
Baidu Index	−0.049 *** (0.013)	−0.037 * (0.020)
GDP		−1.055 *** (0.159)
Population		−0.091 (0.275)
GDP_2		−0.224 (0.246)
IA		0.297 *** (0.044)
Constant	−6.120 *** (0.292)	12.873 *** (2.350)
Time Effect	YES	YES
City Effect	YES	YES
Sample Size	1211	1211
R-Squared	0.854	0.879

Note: Baidu is the most widely used search engine in China; *** and * indicate the significance levels of 1% and 10%, respectively; province-year clustered standard errors are in brackets below the coefficients.
